# Epigenetics and Mitochondrial Stability in the Metabolic Memory Phenomenon Associated with Continued Progression of Diabetic Retinopathy

**DOI:** 10.1038/s41598-020-63527-1

**Published:** 2020-04-20

**Authors:** Renu A. Kowluru, Ghulam Mohammad

**Affiliations:** Kresge Eye Institute, Wayne State University, Detroit, MI USA

**Keywords:** Cell biology, Endocrinology

## Abstract

Retinopathy continues to progress even when diabetic patients try to control their blood sugar, but the molecular mechanism of this ‘metabolic memory’ phenomenon remains elusive. Retinal mitochondria remain damaged and vicious cycle of free radicals continues to self-propagate. DNA methylation suppresses gene expression, and diabetes activates DNA methylation machinery. Our aim was to investigate the role of DNA methylation in continued compromised mitochondrial dynamics and genomic stability in diabetic retinopathy. Using retinal endothelial cells, incubated in 20 mM glucose for four days, followed by 5 mM glucose for four days, and retinal microvessels from streptozotocin-induced diabetic rats in poor glycemia for four months, followed by normal glycemia for four additional months, DNA methylation of mitochondrial fusion and mismatch repair proteins, *Mfn2* and *Mlh1* respectively, was determined. Retinopathy was detected in trypsin-digested microvasculature. Re-institution of good glycemia had no beneficial effect on hypermethylation of *Mfn2* and *Mlh1* and retinal function (electroretinogram), and the  retinopathy continued to progress. However, intervention of good glycemia directly with DNA methylation inhibitors (Azacytidine or Dnmt1-siRNA), prevented *Mfn2* and *Mlh1* hypermethylation, and ameliorated retinal dysfunction and diabetic retinopathy. Thus, direct regulation of DNA methylation can prevent/reverse diabetic retinopathy by maintaining mitochondrial dynamics and DNA stability, and prevent retinal functional damage.

## Introduction

Diabetic retinopathy is the fifth most common cause of preventable blindness, and hyperglycemia and the duration of diabetes are clinically important risk factors for its development and progression. Pivotal Diabetes Control and Complications Trial (DCCT), and the follow-up Epidemiology of Diabetes Interventions and Complications (EDIC), studies have shown that the clinical features of retinopathy continue to develop long after intensive glucose control is maintained in patients treated with the standard treatment regimen during the DCCT. These studies have further demonstrated that intensive control in the early stages of diabetes is critical as its benefits persist beyond the period of its institution, suggesting a ‘metabolic memory’ phenomenon^1,2^. Both *in vitro* and *in vivo* experimental models of diabetic retinopathy also have duplicated this memory phenomenon; retinal histopathology initiated during prior poor glycemic control in dogs and rats does not benefit from the good glycemic control which follows it^3,4^. However, the molecular mechanism of the metabolic memory phenomenon still remains elusive.

Mitochondrial integrity is critical for cell survival, and in diabetes, damaged mitochondria leak cytochrome C, accelerating retinal capillary cell apoptosis, a phenomenon which precedes the formation of acellular capillaries and pericyte ghosts^5–7^. Mitochondria are also highly dynamic, and undergo continuous fusion and fission^8,9^. Fission helps remove damaged mitochondria, and fusion unites two mitochondria, mixing their contents and diluting damaged mitochondrial proteins and DNA (mtDNA). In diabetic retinopathy, mitochondrial dynamics and biogenesis are compromised^10,11^. Mitochondrial fusion protein, mitofusin 2 (Mfn2), is decreased, and  fission protein dynamin 1-like protein (Drp1) is increased, leading to smaller sized mitochondria that have increased mitochondrial DNA (mtDNA) instability^12–14^. In addition, mtDNA itself is damaged, and the damage is more extensive at its D-loop, the region with critical transcription and replication sites^11,15,16^. Sequence variants are significantly increased in the already heteroplasmic mtDNA, and the bad situation is further worsened by suboptimal levels of the mtDNA repair enzyme MutL homolog 1, Mlh1, which is responsible to cut these mismatches^17^. Transcription of mtDNA is impaired and the electron transport chain (ETC) system is compromised^18^, compromising the overall stability of the mitochondria. Our previous work has shown that reinstitution of good glycemic control after a period of poor glycemic control in rats, fails to reverse diabetes-induced mitochondrial damage and decrease in Mfn2 and Mlh1, and mitochondria remain swollen with loosely packed cristae and increased number of sequence variants in its DNA. The compromised ETC system continues to fuel into the vicious cycle of free radicals^12,17^. The mechanism responsible for continual inhibition of Mfn2 and Mlh1, however, remains unclear.

Gene transcription is also regulated by external factors, without altering the DNA sequence, and these epigenetic changes can be erased, or be passed to the next generation^11,19–21^. In diabetes, the machinery responsible for maintaining DNA methylation status including DNA methyl transferases (Dnmts) and Ten-Eleven translocases, is activated in the retina and its vasculature. 5-methyl cytosine (5mC) levels are elevated in the mtDNA, and the promoter DNA of *Mfn2* and *Mlh1* are also hypermethylated. Furthermore, DNA methylation machinery continues to function aberrantly even when the hyperglycemic insult is removed^17,22^. The role of epigenetics in mitochondrial structural and genomic stability in the resistance of retinopathy to reverse after re-institution of normal glycemia remains to be investigated.

The aim of this study was to investigate the molecular mechanism of sustained compromised mitochondrial dynamics and mtDNA stability in retinal microvasculature even when normal glycemia in re-instituted, especially focusing on the role of epigenetics. Using human retinal endothelial cells (HRECs) in culture, and the rat model of diabetic retinopathy, we have investigated the effect of re-institution of normal glycemia on epigenetic modifications of *Mfn2* and *Mlh1*. We have also examined the effect of intervention of the reversal phase with inhibitors of Dnmts on the mitochondrial structural and genomic stability, and on the development of diabetic retinopathy.

## Results

### Retinal endothelial cells

**Figure 1:**
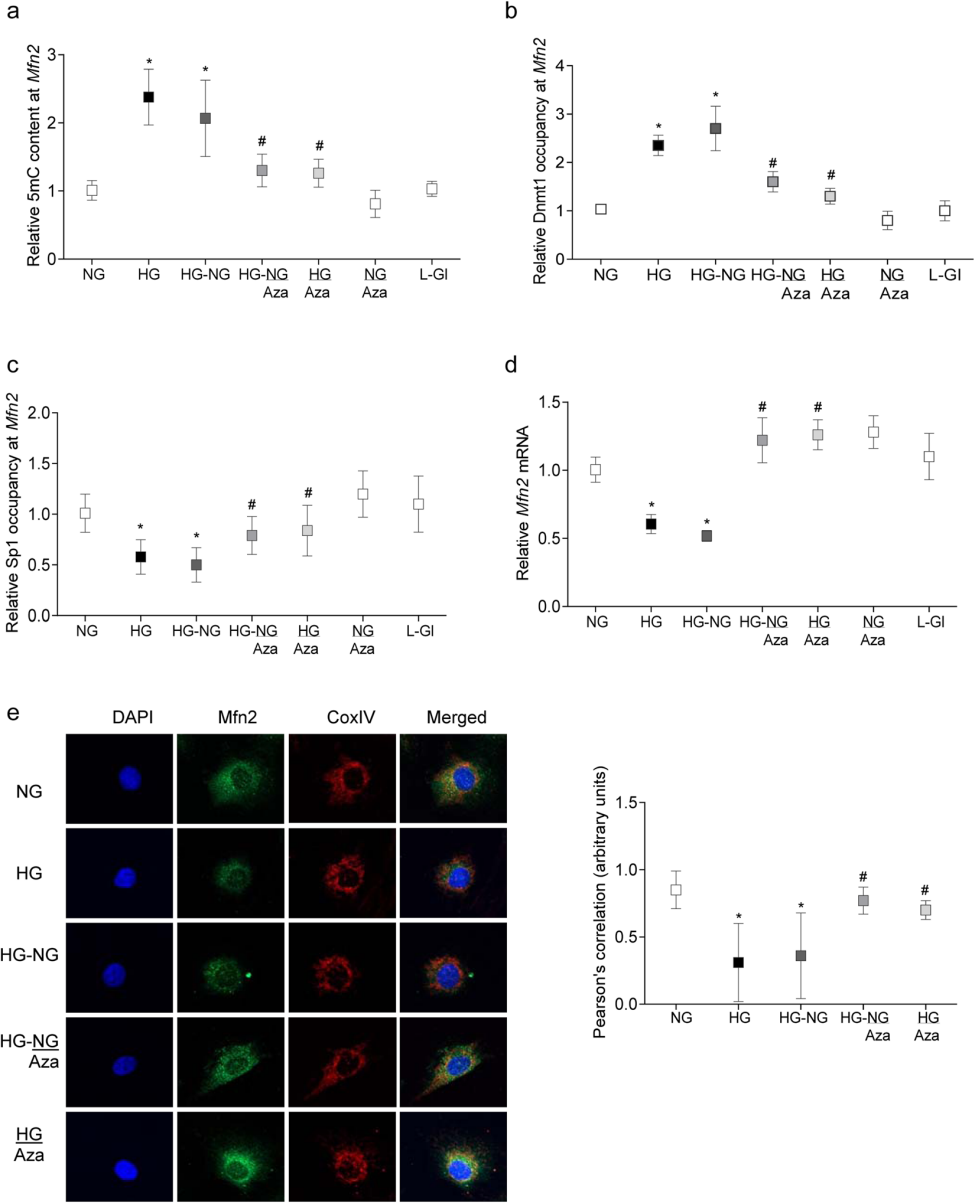
Sustained hypermethylation of *Mfn2* promoter DNA in retinal endothelial cells after removal of high glucose, and intervention with Aza. HRECs incubated in 20 mM glucose (HG) for four days, followed by 5 mM D-glucose (NG) for four days, in the presence or absence of 1 µM Aza were analyzed for (**a**) methylated cytosine at *Mfn2* promoter DNA by Methylated DNA capture method and (**b,c**) Dnmt1 and Sp1 binding by ChIP technique. The values are plotted relative to those obtained from  the ‘input DNA’ internal control. (**d**) *Mfn2* mRNA was quantified by qPCR, and the values are represented as mRNA of *Mfn2*, relative to that of *β-actin*, in each sample. Values from NG group values are adjusted to 1. (**e**) Mitochondrial localization of Mfn2 by immunofluorescence technique using Alexa-Flour 488 (green) and Texas Red (red) conjugated secondary antibodies for Mfn2 and CoxIV respectively. The images were captured in ZEISS 40X objective magnification with Apotome module. Pearson’s correlation coefficient was determined using co-localization software module. Data are represented as mean ± SD of the values obtained from four cell preparations, and each measurement done in duplicate. HG-NG and HG-NG/Aza = HRECs in 20 mM D-glucose for four days followed by 5 mM glucose, without or with Aza respectively, for four additional days; HG/Aza and NG/Aza= HRECs in continuous 20 mM or 5 mM glucose respectively, in the presence of Aza; L-Gl = 20 mM L-Glucose. *and ^#^p < 0.05 compared to NG or HG respectively.

Reversal of hyperglycemic insult fails to ameliorate diabetes-induced increase in Dnmt1^23^, and decrease in Mfn2^12^. Role of epigenetics in sustained decrease in *Mfn2* was investigated by analyzing DNA methylation status of its promoter. As shown in Fig. 1a, compared to cells in normal glucose (5 mM D-glucose, NG), high glucose (20 mM D-glucose, HG) increased 5mC levels at *Mfn2* promoter by 2.5 fold, and 5 mC remained elevated even after removal of high glucose. Similarly, binding of Dnmt1 at *Mfn2* promoter remained elevated, and that of Sp1 decreased (40–50%), in the cells exposed to high glucose for four days, followed by normal glucose for four days (HG-NG group) (Fig. 1b,c). Consistent with sustained hypermethylation of *Mfn2* promoter, gene transcripts of *Mfn2* also   remained  compromised in HG-NG group (Fig. 1d). Compared with cells in normal glucose, Mfn2 expression in the mitochondria was significantly reduced in HG and HG-NG groups (Fig. 1e); the accompanying graph shows ~50% lower Pearson’s correlation in HG and HG-NG groups vs NG group. Values obtained from the cells in 20 mM L-glucose (osmotic/metabolic control, L-Gl) were not different from those obtained from normal glucose.

**Figure 2:**
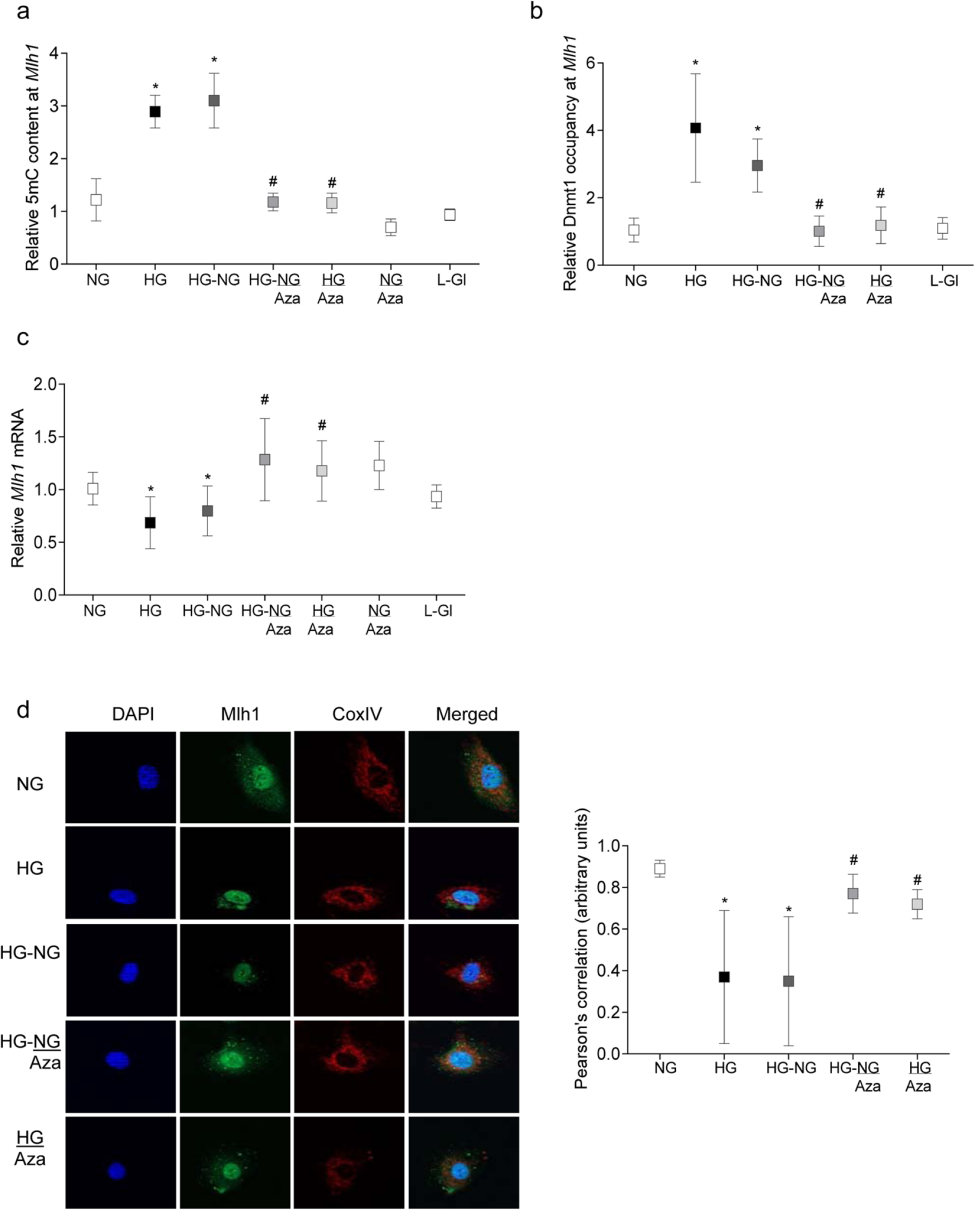
*Mlh1* promoter DNA hypermethylation and reversal of high glucose insult. HRECs in 20 mM D-glucose for four days, followed by four days in 5 mM D-glucose, with or without 1 µM Aza were utilized for quantifying (**a**) relative 5 mC content at *Mlh1* promoter by MeDIP, (**b**) Dnmt1 binding by ChIP and (**c**) *Mlh1* mRNA by qPCR using β-actin as a housekeeping gene. The values in NG group are adjusted to 1. (**d**) Mlh1 mitochondrial localization was determined by immunofluorescence technique. Pearson’s correlation coefficient was calculated using co-localization software module. Results are represented as mean ± SD from four cell preparations, with each measurement made in duplicate or triplicate. *p < 0.05 compared to NG, and ^#^p < 0.05 compared to HG.

Stability of mtDNA is also impaired in diabetes, and re-institution of good glycemic control fails to provide any benefit to the sequence mismatches in the mtDNA, induced by prior poor glycemic control^17^. To understand the role of epigenetics in sustained poor mtDNA quality, DNA methylation status of *Mlh1* promoter was investigated. Glucose-induced increase in 5mC and Dnmt1 binding at *Mlh1* promoter failed to reverse even after four days of normal glucose, and *Mlh1* gene transcripts remained subnormal (Fig. 2a–c). In addition, compared to NG group, immunofluorescence data, followed by Pearson correlation, showed continued decreased expression of Mlh1 in the mitochondria in HG and HG-NG groups.

#### Supplementation with Dnmt inhibitor during reversal of high glucose insult

Intervention of the reversal phase with the inhibitors of Dnmt restores mtDNA methylation, and prevents decrease in the transcription of mtDNA-encoded genes^23^. The effect of inhibition of Dnmts on restoration of mitochondrial dynamics and DNA stability was investigated. Supplementation of four days of normal glucose, which had followed four days of high glucose, with 1 µM 5-aza-2′-deoxycytidine (Aza, HG-NG/Aza group), restored DNA methylation and binding of Dnmt1 and Sp1 at *Mfn2* promoter to the normal levels. The values were significantly different from those observed in HG or HG-NG groups (Fig. 1a–c). In the same samples, decrease in *Mfn2* gene transcripts was also restored (Fig. 1d). Since the translocation of Mfn2 inside the mitochondria is important to maintain their dynamics^12^, effect of Aza supplementation during reversal phase on Mfn2 accumulation inside the mitochondria was investigated. Aza significantly improved Mfn2 mitochondrial localization as confirmed by increased Mfn2-CoxIV co-localization, and higher Pearson’s correlation in HG/Aza and HG-NG/Aza groups compared to HG and HG-NG groups (Fig. 1e).

Addition of Aza, either from the beginning of high glucose exposure (HG/Aza), or during the reversal phase, also reinstated DNA methylation status of *Mlh1* promoter; 5 mC levels and Dnmt1 binding at its promoter were similar to those obtained from cells in continuous normal glucose. Similarly, Mlh1 expression and mitochondrial accumulation were also significantly higher compared to the cells in HG or HG-NG groups (Fig. 2a-d).

**Figure 3:**
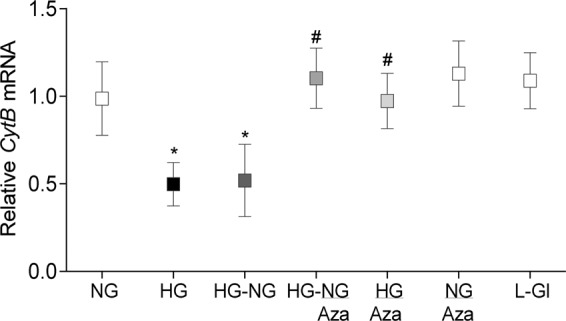
Direct inhibition of Dnmts during reversal of high glucose insult and mtDNA transcription. Gene transcripts of mtDNA-encoded *CytB* was quantified in HRECs incubated in high glucose for four days, followed by normal glucose for four days, in the presence or absence of 1 µM 5-aza-2′-deoxycytidine (Aza) by qPCR; β-actin was used as a housekeeping gene. Results are presented as mean ± SD of the values obtained from at least three preparations, with each measurement performed in triplicate. HG-NG and HG-NG/Aza = 20 mM D-glucose for four days followed by 5 mM glucose, without or with Aza respectively, for four additional days; HG/Aza and NG/Aza= HRECs in continuous 20 mM or 5 mM D-glucose respectively, in the presence of Aza; L-Gl = 20 mM L-Glucose. * and ^#^p < 0.05 compared to NG or HG respectively.

Mlh1 plays a critical role in DNA mismatch repair process^17^, and defects in mitochondrial fusion are also associated with accumulation of mtDNA point mutations and deletions^24^; intervention of the reversal phase with Aza on mtDNA stability was confirmed by quantifying the transcripts of mtDNA-encoded *CytB* in the same cell preparations. As shown in Fig. 3, Aza restored transcription of *CytB;* the values in HG-NG/Aza and HG/Aza were similar to those in NG or L-Gl groups.

### Rat model of metabolic memory

The role of epigenetics in the continued progression of diabetic retinopathy was confirmed in a rat model of diabetic retinopathy. Rats in continuous poor glycemic control for eight months (PC group) had consistently lower body weight and elevated blood sugar and 24 hour urine output compared to their age-matched normal rats (Norm). Rats in continuous good glycemic control (GC) and Norm groups had similar body weights, blood glucose and urine volumes. Body weights, blood glucose and urine volumes of PC-GC group rats (poor control for four months, followed by good glycemia for four additional months), during their four months of poor glycemia were similar as in PC group. After four months of good glycemia, their body weights became similar to those in GC or Norm group. Rats receiving Aza or *Dnmt1*-siRNA, either throughout the eight months of poor glycemia (PC/Aza or PC/*D*-si groups), or during the four months of good glycemia (PC-GC/Aza, PC-GC/*D*-si groups), had similar body weight, blood glucose and urine volumes as in PC and PC-GC groups respectively, suggesting that Aza or *Dnmt1*-siRNA had no effect on the severity of hyperglycemia (Table 1).

**Table 1:** Metabolic parameters of rats in various hyperglycemic controls.

Groups	Body weight (g)	Blood glucose (mg/dl)	Urine volume 24 hour (ml)
Normal	566 ± 33	108 ± 4	15 ± 2
PC	373 ± 88*	528 ± 7*	164 ± 27*
PC/Aza	362 ± 42*	507 ± 96*	93 ± 41*
PC/*D*-si	381 ± 60*	501 ± 108*	115 ± 52*
PC-GC	348 ± 26*→ 495 ± 22^#^	482 ± 95→ 105 ± 5^#^	121 ± 22*→ 21 ± 6^#^
PC-GC/Aza	349 ± 40*→ 502 ± 22^#^	488 ± 111→ 106 ± 11^#^	125 ± 57*→ 15 ± 9^#^
PC-GC/*D*-si	358 ± 39*→ 582 ± 45^#^	571 ± 61→ 118 ± 26^#^	140 ± 35*→ 19 ± 6^#^
GC	582 ± 27^#^	103 ± 7^#^	13 ± 7^#^

Measurements were made in 7–9 rats/ group, and the values are represented as mean ± SD. PC = Rats maintained in continuous poor glycemic control for eight months; PC/Aza and PC/*D*-si = rats receiving Aza or *Dnmt1*-siRNA respectively, throughout their eight months of continuous poor glycemic control; PC-GC = rats in poor control for four months followed by four months of good control; PC-GC/Aza and PC-GC/*D*-si = Rats in poor control for four months, followed by in good control supplemented with Aza or *Dnmt1*-siRNA respectively, for four months; GC = Rats in continuous good glycemic control for eight months. * and ^#^p < 0.05 compared to Normal or PC respectively.

**Figure 4:**
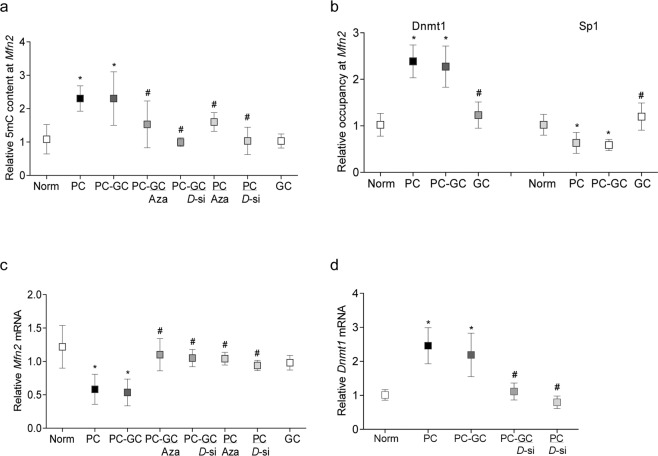
Effect of Dnmt inhibition during good glycemic control in rats, which had followed poor glycemic control, on *Mfn2*. Retinal microvessels from diabetic rats in poor glycemic control for four months, followed by four additional months of good glycemic control, supplemented with Aza or *Dnmt1*-siRNA, were analyzed for relative (**a**) 5 mC levels at *Mfn2* promoter by MeDIP and (**b**) binding of Dnmt1 and SP1 by ChIP technique, using input DNA as an internal control. (**c,d**) *Mfn2* and *Dnmt1* mRNA levels were quantified by qPCR using β-actin as a housekeeping gene. Values are represented as mean ± SD obtained from six to eight animals in each group and normal rat values are adjusted to 1. Norm = Normal; PC, PC/Aza and PC/*D*-si = Rats in poor continuous control for eight months receiving no supplement or Aza or *Dnmt1*-siRNA respectively; PC-GC, PC-GC/Aza and PC-GC/*D*-si = Rats in poor control for four months followed by four months of good control, receiving no supplement or Aza or *Dnmt1*-siRNA respectively; GC = Rats in continuous good glycemic control for eight months. *and ^#^p < 0.05 compared to Norm or PC respectively.

Re-institution of good glycemic control in previously poor glycemic control rats had no effect on the hypermethylation of *Mfn2* promoter DNA, and compared to rats in Norm group, four months after initiation of good glycemic control in previously poor control rats, 5 mC and Dnmt1 binding remained 2–2.5 fold elevated with ~50% decrease in Sp1 binding (Fig. 4a,b). This was accompanied by sustained decrease in *Mfn2*, and increase in *Dnmt1*, gene transcripts in the same retinal microvessel preparations from PC-GC group rats (Fig. 4c,d).

**Figure 5:**
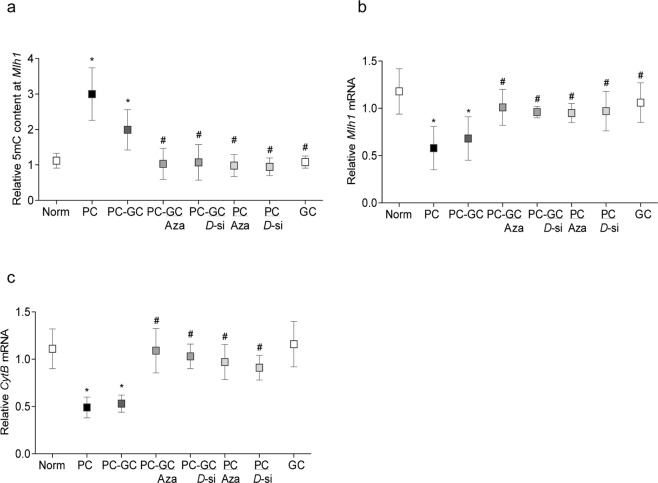
Regulation of Dnmts and *Mlh1* transcription and mitochondrial damage. (**a**) Relative 5mC levels at *Mlh1* promoter and (**b**) *Mlh1* mRNA and (**c**) *CytB* mRNA (mitochondrial damage) were quantified in retinal microvessels. Each group represents data from six or more rats, with measurements made in duplicate or in triplicate. *p < 0.05 compared to Norm, and ^*#*^p < 0.05 compared to PC.

As with Mfn2, re-institution of normal glycemia did not produce any beneficial effects on *Mlh1*, and its promoter continued to be hypermethylated and gene transcripts suppressed (Fig. 5a,b).

Re-institution of good glycemic control, soon after induction of diabetes in rats (GC group), however, protected hypermethylation of *Mfn2* and *Mlh1* promoter DNA, and along with the binding of Dnmt1, prevented decrease in their gene transcripts. The values in GC group were not different from those obtained in Norm group (Figs. 4 and 5).

#### Intervention of reversal phase with DNA methylation inhibitors

Mitochondrial structural and functional stability: Administration of Aza or *Dnmt1*-siRNA, during the four months of good glycemic phase, which had followed the four months of poor glycemia (PC-GC/Aza or PC-GC/*D*-si), or soon after induction of diabetes (PC/Aza or *D*-si), reversed or prevented hypermethylation of *Mfn2* promoter and decrease in its gene transcripts. The values in PC and PC-GC groups were significantly different than those obtained in PC/Aza or PC/*D*-si and PC-GC/Aza or PC-GC/*D*-si groups (Fig. 4a–c). In the same group of rats, *Dnmt1*-siRNA administration also attenuated diabetes-induced increase in *Dnmt1* gene transcripts (Fig. 4d).

Consistent with *Mfn2*, Dnmt inhibitors also restored hyperglycemia-induced elevation in 5mC at *Mlh1* promoter DNA and decrease in its gene transcripts; the values in PC-GC/Aza or PC-GC/*D*-si or PC/Aza or PC/*D*-si groups were not different from those in Norm or GC groups (Fig. 5a,b). Likewise, mtDNA-encoded *CytB* transcripts in PC-GC/Aza or PC-GC/*D*-si group were significantly higher compared to PC or PC-GC group (Fig. 5c).

**Figure 6:**
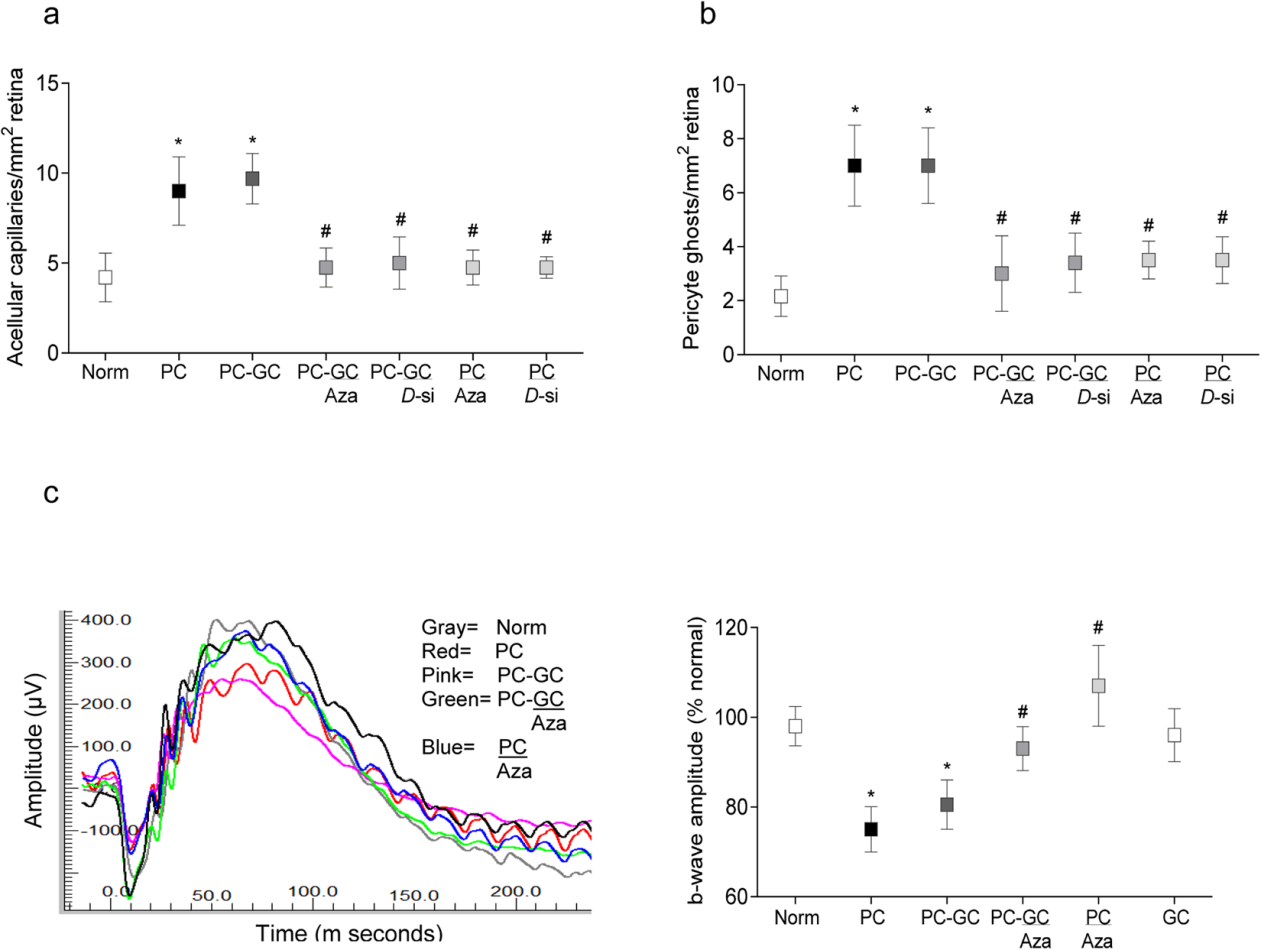
Protection, and reversal, of diabetic retinopathy and impaired retinal function by inhibition of Dnmts. (**a,b**) Trypsin-digested retinal microvessels, stained with periodic acid-Schiff-hematoxylin, were analyzed for degenerative capillaries and pericyte ghosts. (**c**) Five to eight days before termination of the experiment, ERG was performed in dark-adapted (overnight) rats by Ocuscience HMsERG system, and b-wave amplitude at 10,000 mcd.s/m^2 was calculated. The values are mean ± SD from six to nine rats/group. Norm = Normal; PC, PC/Aza and PC/*D*-si = Continuous poor control for eight months without any supplement, or Aza or *Dnmt1*-siRNA respectively; PC-GC, PC-GC/Aza and PC-GC/*D*-si = Poor control for four months followed by good control for four months without any supplement, or Aza or *Dnmt1*-siRNA respectively; GC = Continuous good glycemic control for eight months. *and ^#^p < 0.05 compared to Norm or PC respectively.

Retinopathy and retinal function: Re-institution of good glycemic control after a period of poor glycemic control in rats, as expected, had no effect on retinal histopathology associated with diabetic retinopathy, the number of acellular capillaries and pericyte ghosts remain elevated in PC and PC-GC groups (Fig. 6a,b). Retinal function was also impaired in PC and PC-GC groups; the amplitude of b-wave was decreased by ~20% in these rats, compared to the rats in Norm group (Fig. 6c).

However, supplementation with Aza or *Dnmt1*-siRNA, during the good glycemic phase, which had followed poor glycemia, or soon after induction of diabetes, ameliorated retinal pathology. Compared to PC-GC or PC groups, acellular capillaries and pericyte ghosts in Aza or *Dnmt1*-siRNA-treated rats (PC-GC/Aza or PC-GC/*D*-si, and PC/Aza or PC/*D*-si groups) were significantly lower, and these values were not different from those obtained from rats in Norm or GC groups (Fig. 6a,b). Consistent with retinal vascular histopathology, Dnmt inhibitors also restored retinal function. As shown in Fig. 6c, ~20% decrease in the amplitude of b-wave in PC and PC-GC groups compared to Norm group, was not observed in Aza treated rats (PC-GC/Aza or PC/Aza groups).

## Discussion

Mitochondria are dynamic organelles, and their functional integrity is crucial for the maintenance of cellular homeostasis. In diabetes, mitochondria are damaged and their dysfunction is considered to play a major role in the pathogenesis of diabetic retinopathy^10,11^. Although hyperglycemia is considered as the main integrator of metabolic abnormalities associated with diabetic retinopathy, re-institution of intensive glycemic control after a period of hyperglycemic insult does not cease progression of retinopathy^1,2,25^. Mitochondria stay dysfunctional and their DNA remains damaged with suboptimal transcription of mtDNA-encoded genes. The dysfunctional mitochondria continue to fuel into the vicious cycle of free radicals^18,26,27^. Furthermore, the activation of the machinery responsible for DNA methylation does not benefit from re-institution of intensive glycemic control, and hypermethylation of mtDNA and hydroxymethylation of *MMP-9* promoter continues^21–23^. Here, we provide convincing data showing the role of DNA methylation in sustained compromised mitochondrial structural and DNA stability. *Mfn2* and *Mlh1* promoters remain hypermethylated with increased Dnmt1 binding, and accumulation of Mfn2 and Mlh1 in the mitochondria remains suboptimal. However, intervening the good glycemic phase, which has followed the poor glycemia, directly with the inhibitors of DNA methylation (pharmacological or molecular), prevents hyperglycemia-induced increase in *Mfn2* and *Mlh1* DNA methylation, and restores their gene expressions and mitochondrial accumulation. Consistent with DNA methylation, this is also the first *in vivo* study showing that supplementation of Dnmt inhibitors soon after induction of diabetes, or intervention with them during the reversal phase, prevents/ceases progression of diabetic retinopathy. The results demonstrate the role of DNA methylation in the development of diabetic retinopathy and in the metabolic memory associated with its continued progression.

Mitochondria are structurally dynamic, and to maintain their function they constantly fuse and divide changing their size and shape^9,28^. An imbalance in fusion-fission alters overall mitochondrial morphology; while disruption of fusion results in mitochondria to fragment into short rods or spheres, that of fission generates elongated, interconnected tubules^29^. Mitochondrial dynamic also controls many physiological processes including their membrane potential, respiration and genomic stability. Although the loss of mitochondrial fission has no deleterious effect on mtDNA levels, defects in fusion are associated with the accumulation of mtDNA point mutations and deletions, resulting in compromised ETC system^24^. Mitochondrial DNA harbors both mutant and non-mutant DNA within the same cell, but if there is significant increase in the mutant mtDNA, cells exhibit reduced energy capacity^30^. Increase in mtDNA sequence variants have been associated with many chronic diseases including open angle glaucoma^31,32^. The number of sequence variants is increased in the retinal mtDNA in diabetes, and Mlh1 expression is decreased^17,22^. Our recent work has shown that due to hypermethylation of the promoter DNA of *Mfn2* and *Mlh1*, their gene transcripts are downregulated, mtDNA mismatches are increased and mitochondrial function is compromised^13,14^. Here, we clearly demonstrate that the institution of intensive control fails to provide any benefit to DNA hypermethylation of both *Mfn2* and *Mlh1* promoters, and mtDNA transcription remains impaired.

DCCT-EDIC studies have shown that institution of early intensive therapy lowers the incidence of further progression of retinopathy, and even 30 years after the start of DCCT, former intensive therapy participants still report better Visual Function than the former conventional group^33^. Animal models have also demonstrated that institution of good glycemic control, soon after establishment of diabetes, prevents impairments in mitochondrial biogenesis, and the development of retinopathy^15,17^. Here, our data from GC group show that *Mfn2* and *Mlh1* promoters are also spared from epigenetic modifications, further supporting the significance of early and continued intensive glycemic control for a diabetic patient.

DNA methylation profiling of genomic DNA of whole blood isolated at EDIC Study baseline, and at year ten, show that DNA-methylation differences during the DCCT persist at certain loci associated with glycemia for several years during the EDIC Study, supporting an epigenetic explanation for metabolic memory^34^. Our results from both *in vitro* and *in vivo* models presented here clearly show that direct inhibition of Dnmts during the good glycemic phase, which has followed poor glycemic phase, restores *Mfn2* and *Mlh1* DNA methylation and their gene transcription. This further confirms the role of epigenetics in sustained downregulation of mitochondrial structural and DNA stability. Inhibitors of DNA methylation also prevent the development of diabetic retinopathy, as seen by similar number of retinal degenerative capillaries and pericyte ghosts in Norm and PC/Aza groups. This further supports the possible role of DNA methylation in the development of diabetic retinopathy. Supplementation with lipoic acid, a direct scavenger of superoxide, during the good glycemic control, which has followed poor control, has beneficial effect on the impaired retinal mitochondria biogenesis and the development of diabetic retinopathy in a rat model of metabolic memory^15^. Results presented here clearly show that direct inhibition of DNA methylation during the good glycemic phase, which had followed poor glycemic phase, also ceases progression of retinopathy as evidenced by decreased number of acellular capillaries and pericyte ghosts in PC-GC/Aza and PC-GC/*D*-si groups. This suggests that if Dnmt inhibitors are supplemented (four months of hyperglycemic) before retinal capillary cells begin to undergo accelerated apoptosis, further DNA methylation of the genes important in maintaining mitochondrial homeostasis is prevented. This could prevent mtDNA damage-electron transport chain dysfunction, and interfere with the vicious cycle of free radicals.

We recognize that DNA methylation is critical in maintaining gene transcription^35^, and the possibility that supplementation with Aza or *Dnmt1*-siRNA could have some unwarranted effects on other genes important in mitochondrial homeostasis, or associated with many other metabolic abnormalities implicated in the development of diabetic retinopathy^22,36,37^, cannot be ruled out. Furthermore, the route of administration of Dnmt inhibitors in rats (*Dnmt1*-siRNA- intravitreal or Aza- intraperitoneal) does not discriminate the specific retinal cell types being targeted, beneficial effects of these inhibitors on retinal vascular histopathology and ERG changes, however, clearly imply that vascular and neural cells are some of the retinal cell types for their actions. This is further supported by similar results from isolated retinal endothelial cells in culture. There also remains a possibility that reduction in further progression of diabetic retinopathy by Aza or *Dnmt1*-siRNA supplementation during the reversal phase could be due to regulation of mtDNA methylation itself. Our data showing beneficial effects of regulation of Dnmts on mitochondrial fusion and mismatch repair machinery, and retinal histopathology, suggest an important role of DNA methylation in the metabolic memory phenomenon.

**Figure 7:**
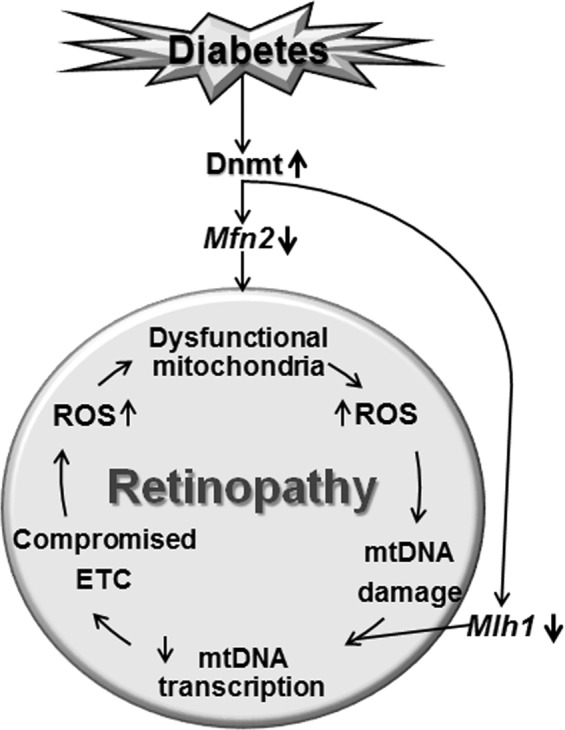
Working model. Mitochondria are damaged in diabetes, and ETC system becomes compromised, and continuous production of free radicals damages their DNA, impairing its transcription. This further compromises the ETC system, and the vicious cycle of free radicals continues to self-propagate, giving no break to the already damaged mitochondria. Dnmts are also redox-sensitive enzymes, and due to their sustained activation, *Mfn2* and *Mlh1* remain hypermethylated (and suppressed), further fueling into the vicious cycle, and the retinopathy continues to progress.

In conclusion, our results using experimental models demonstrate the role of DNA methylation in continued mitochondrial functional and genomic stability. Hyperglycemic milieu activates Dnmts, hypermethylating of *Mfn2* and *Mlh1* promoters. Decreased levels of Mfn2 continue to impair mitochondrial dynamics, and suboptimal mismatch repair system does not give a break to the transcription of mtDNA. Compromised ETC continues to fuel into the vicious cycle of free radicals, leaving mitochondria dysfunctional, and the retinopathy continues to progress (Fig. 7). Further, direct regulation of DNA methylation prevents/halts diabetic retinopathy by maintaining mitochondrial dynamics and mtDNA stability, highlighting possible additional opportunities for patients to ameliorate/halt progression of diabetic retinopathy during the early stages of the disease. Supplementation of the best sensible glycemic control with the therapies targeted towards regulating DNA methylation-mitochondria homeostasis could be a welcoming sign for diabetic patients to prevent/arrest progression of this blinding disease.

## Methods

### Retinal endothelial cells

Human retinal endothelial cells (Cell system, Kirkland, WA) from 7–8^th^ passage were incubated in NG or HG media containing 1% fetal calf serum, 9% Nu-serum and 1 µg/mL endothelial growth supplement for 4 days^13,14^. Cell incubated in high glucose were divided into two groups, cells in group 1 remained in high glucose for eight days, in the absence or presence of 1 µM 5-aza-2′-deoxycytidine (Aza; Sigma-Aldrich Corp, St. Louis, MO, USA), respectively. In group 2, after four days of high glucose exposure, the cells were incubated in normal glucose for four additional days, in the absence or presence of Aza (HG-NG and HG-NG/Aza). For osmotic/ metabolic control, 20 mM D-glucose was replaced by 20mM L-glucose (L-Gl). As reported previously, under these incubation conditions, morphology of HRECs was maintained^22^.

### Rats

Male Wistar rats (200–220 g) were obtained from Harlan Labs (South Easton, MA, USA). Diabetes was induced by streptozotocin injection (55 mg/Kg BW, i.p., Sigma-Aldrich), and soon after establishment of diabetes, these rats were divided into five groups. Rats in group 1 remained in poor glycemic control (PC, glycated hemoglobin GHb ~12%) for eight months, group 2 rats received Aza (PC/Aza group, 10 mg/kg/BW, i.p, in sterile PBS) every other day^38^, and in group 3 rats received *Dnmt1*-siRNA, once every 30 days (PC/*D*-si group), as described previously^14^. Briefly, rats anesthetized with ketamine- xylazine were injected intravitreally via a 32 gauge needle in the left eye under a dissecting microscope 10 μg *Dnmt1-*siRNA (Cat. No. ID:RSS331349, Thermo Fisher Scientific, Waltham, MA), mixed with 5 μl Invivofectamine (Cat. No. IVF 3001, Invitrogen, Carlsbad, CA). The right eye received 5 μl medium GC content siRNA negative control (Cat. No. 12935–300, Thermo Fisher Scientific). The efficiency of *Dnmt1*-siRNA was ~50% as determined by retinal gene and protein expressions of Dnmt1. Published reports by others, and our previous work, have also shown that once/month intravitreal administration of siRNA is effective in regulating their target genes^14,39^. Rats in group 4 were in poor glycemic control for four months followed by good glycemic control (GHb ~6%) for another four months (PC-GC group), and in group 5, the rats remained in good glycemic control for the entire eight months (GC). Good glycemia was maintained by injecting a total of 5–7 IU insulin (Humulin N; Eli Lilly, Indianapolis, Indiana), two times/day; 2–4 IU in the morning (7–8am) and 3–4 units in the evening (5:30–7 pm). This regimen, and the dose, maintains rats in normoglycemia, without resulting in any major hypoglycemic shock^17,27^. Just before initiation of good glycemic control (after four months of poor glycemic control), rats in group 4 were further divided into three subgroups, while subgroup 1 rats remained in good glycemic control for four months (PC-GC), subgroup 2 rats received Aza during these four months of good control (PC-GC/Aza group), and subgroup 3 rats received *Dnmt1*-siRNA intravitreally every 30 days (PC-GC/*D*-si). Four months of poor glycemic control, followed by four months of good glycemia, was used because at four months of hyperglycemia, although some mtDNA damage could be observed, capillary cell apoptosis and histopathology of diabetic retinopathy are not detectable. However, at six to eight months of hyperglycemia, mtDNA damage, capillary cell apoptosis and retinal histopathology are clearly observed^40,41^. Age-matched normal rats were used as controls (Norm). Each of the eight experimental groups (Norm, PC, GC, PC/Aza, PC/*D*-si, PC-GC, PC-GC/Aza, PC-GC/*D*-si) had 8–10 rats. Rats were housed in metabolic cages throughout the study, and their 24-hour urine samples were collected once a week. Rats were weighed two to three times/week, and their blood glucose was measured every seven to ten days (Glucometer Elite, Bayer Corporation)^17,27^. At the end of the experiment, the rats were euthanized by carbon dioxide asphyxiation and their retina was isolated immediately, or the eyeball was stored in formalin (for trypsin digestion). The treatment of the animals followed the guidelines of the Association for Research in Vision and Ophthalmology Resolution on the Use of Animals in Research, and the experimental protocols were approved by Wayne State University’s Animal Care and Use Committee.

Freshly isolated retina from these rats in various metabolic control, as described above, was employed to prepare microvessels by incubating it in 10–15 ml distilled water for one hour at 37 °C^14,42^, followed by gentle removal of the nonvascular tissue under a microscope.

### Gene expression

Trizol extracted total RNA was used for cDNA synthesis employing a High Capacity cDNA Reverse Transcription kit (Applied Biosystems, Foster City, CA). SYBR green based Real-time quantitative PCR (qPCR) was performed in ABI 7500 Cycler detection system (Applied Biosystems) using gene- and species- specific primers (Table 2). β-actin was employed as a housekeeping gene, and a delta Ct method was employed to calculate relative mRNA expression^14,17^.

**Table 2:** Primer sequences.

Primer	Sequence
**Human**	
*Mfn2*	F-ATGCAGACGGAAAAGCACTT
	R-ACAACGCTCCATGTGCTGCC
*Mfn2* promoter	F-TGCCCGATGAGTCACTTCAC
	R-CAAGGGGCGAAAAACCAAGG
*Mlh1*	F-CCTGCTCCCCGCGCTTTCTT
	R-CGGGGAGGCTGTGCTTCTGC
*Mlh1* promoter	F-GTCATCCACATTCTGCGGGA
	R-CTCTGCTGAGGTGATCTGGC
*CytB*	F-ATGGTAGATGTGGCGGGTTT
	R-TCTCCGATCCGTCCCTAACA
*β-Actin*	F-AGCCTCGCCTTTGCCGATCCG
	R-TCTCTTGCTCTGGGCCTCGTCG
**Rat**	
*Mfn2*	F-ACAAAGTTCTGCCATCTGGG
	R-TGCTCATCCTGATGGAGGGC
*Mfn2* promoter	F-GTCTGCCCGATGAGTCACTT
	R-AAACCAAGGGCGTGGAGTA
*Mlh1*	F-AAGCATAAGCCATGTGGCCC
	R-TTCCCGTACTCTTCACTGGG
*Mlh1* promoter	F- TTCCCGAGTAGAGGCGACC
	R- AACCCAGGGGGGTGCTTGG
*Dnmt1*	F-ACCTACCACGCCGACAT
	R-AGGTCCTCTCCGTACTCCA
*CytB*	F-CCCATTCATTATCGCCGCCC
	R-GGTCTCCTAGTAGGTCTGGG
*β-Actin*	F-CCTCTATGCCAACACAGTGC
	R-CATCGTACTCCTGCTTGCTG

### Methyl cytosine and Dnmt1 binding

Using MethyLamp Methylated DNA capture (MeDIP) Kit (EPIGENTEK, Farmingdale, NY), 5mC was immunoprecipitated in the sonicated total genomic DNA^13,14,22^. Enriched 5mC fractions were quantified by qPCR using gene and species-specific primers (Table 2), and input DNA was used as an internal control. For quantitative analysis of ChIP products, qPCR was carried out to determine enrichment of 5mC in the gene of interest, relative to the input DNA.

Chromatin immunoprecipitation (ChIP) technique was employed to quantify the binding of Dnmt1 or transcription factor Sp1, at the gene promoters employing the methods described previously^17^. Each experiment had input DNA as an internal control and IgG (Cat. No. ab171870, Abcam) as antibody control. Quantitative analysis of ChIP products was performed by qPCR to determine occupancy of Dnmt1 or Sp1 at the gene promoter, relative to that in the input DNA. IgG control values were <0.5% of the values obtained from Dnmt1 or Sp1 antibody.

### Mitochondrial accumulation of Mfn2 and Mlh1

Expression of Mfn2 or Mlh1 in the mitochondria was performed in HRECs by immunofluorescence technique using antibodies against Mfn2 (Cat. No. ab56889, Abcam; 1:250 dilution) and Mlh1 (cat. no. ab92312, Abcam, Cambridge, MA, USA; 1:100 dilution), as previously described^13,14^. CoxIV was used as a mitochondrial marker; Cat. No. ab153709 for Mfn2 and Cat. No. ab33985 for Mlh1; both from Abcam, and at 1:250 dilution each. Secondary antibodies included Alexa Fluor-488 (green) conjugated anti-rabbit (Cat. No. Molecular Probes-Life Technologies, Grand Island, NE), DyLight 488-conjugated anti-mouse (Cat. No. DI-2488, Vector Laboratories, Burlingame, CA) and Texas red-conjugated anti-mouse (Cat. No. TI-2000, Vector Laboratories, Burlingame, CA); each at 1:500 dilution. Immuno-labelled cells were mounted using DAPI-containing (blue) Vectashield mounting medium (Vector Laboratories), and were examined under ZEISS (Carles Zeiss, Inc., Chicago, IL, USA) at 40X objective magnification with the Apotome module^13,14^. The images were calibrated with the ZEISS proinbuilt software package and modules, and the Pearson’s correlation coefficient was calculated using the colocalization software module^13^.

### Retinal histopathology

Retina was isolated from the formalin-fixed eyes, and after rinsing overnight in running water, the retina was incubated for one hour at 37 °C in 3% crude trypsin (Invitrogen-Gibco, Grand Island, NY) supplemented with 200 mM sodium fluoride. The vasculature was cleaned under a microscope, and stained with periodic acid-Schiff-hematoxylin (PAS, Cat No. 395B, Sigma). Degenerative capillaries and pericyte ghosts were counted under a microscope^27,43^.

### Electroretinogram

Rats were dark-adapted overnight, and after anesthetizing with Ketamine-Xylazine, their pupils were dilated by 2.5% phenylephrine and 1% tropicamide ophthalmic solutions. Silver embedded thread eye electrode was placed at the corneal surface through a thin layer of 1% methylcellulose. Needle electrodes were placed in the tail and cheek as ground and reference electrodes respectively. ERG responses were recorded by Ocuscience HMsERG (OcuScience LLC, Kansas City, MO) using Ganzfeld flashes with intensities ranging from 100–25,000 mcd.s/m^2^. The rats were maintained on a heating board (37 °C) throughout the experiment. The amplitude of b-wave was calculated by ERGview4.882 software^43^.

### Statistical analysis

Data are presented as mean ± SD. Group comparisons were performed using one-way ANOVA followed by Dunn’s *t*-test, or by Bonferroni test for multiple comparisons; and p < 0.05 was considered significant.

## Data Availability

R.A.K. is the guarantor of this work and, as such, had full access to all the data in the study and takes responsibility for the integrity of the data and the accuracy of the data analysis.

## References

[1] Aiello LP (2014). Diabetic retinopathy and other ocular findings in the diabetes control and complications trial/epidemiology of diabetes interventions and complications study. Diab Care.

[2] Writing Team for the, D. E. R. G. (2016). Effects of Prior Intensive Insulin Therapy and Risk Factors on Patient-Reported Visual Function Outcomes in the Diabetes Control and Complications Trial/Epidemiology of Diabetes Interventions and Complications (DCCT/EDIC) Cohort. JAMA Ophthalmol..

[3] Engerman RL, Kern TS (1987). Progression of incipient diabetic retinopathy during good glycemic control. Diabetes.

[4] Kowluru RA (2003). Effect of reinstitution of good glycemic control on retinal oxidative stress and nitrative stress in diabetic rats. Diabetes.

[5] Kowluru RA, Abbas SN (2003). Diabetes-induced mitochondrial dysfunction in the retina. Invest. Ophthalmol. Vis. Sci..

[6] Mizutani M, Kern TS, Lorenzi M (1996). Accelerated death of retinal microvascular cells in human and experimental diabetic retinopathy. J. Clin. Invest..

[7] Kowluru RA (2005). Diabetic retinopathy: mitochondrial dysfunction and retinal capillary cell death. Antiox Redox Signal..

[8] Suen DF, Norris KL, Youle RJ (2008). Mitochondrial dynamics and apoptosis. Genes. Dev..

[9] Silva Ramos E, Larsson NG, Mourier A (2016). Bioenergetic roles of mitochondrial fusion. Biochim. Biophys. Acta.

[10] Kowluru RA, Kowluru A, Mishra M, Kumar B (2015). Oxidative stress and epigenetic modifications in the pathogenesis of diabetic retinopathy. Prog. Ret Eye Res..

[11] Kowluru RA (2019). Mitochondrial stability in diabetic retinopathy: Lessons learned from epigenetics. Diabetes.

[12] Zhong Q, Kowluru RA (2011). Diabetic retinopathy and damage to mitochondrial structure and transport machinery. Inves Ophthalmol. Vis. Sci..

[13] Mohammad G, Radhakrishnan R, Kowluru RA (2019). Epigenetic Modifications Compromise Mitochondrial DNA Quality Control in the Development of Diabetic Retinopathy. Invest. Ophthalmol. Vis. Sci..

[14] Duraisamy AJ, Mohammad G, Kowluru RA (2019). Mitochondrial fusion and maintenance of mitochondrial homeostasis in diabetic retinopathy. Biochim. Biophys. Acta.

[15] Santos JM, Tewari S, Goldberg AFX, Kowluru RA (2011). Mitochondria biogenesis and the development of diabetic retinopathy. Free. Rad. Biol. Med..

[16] Tewari S, Santos JM, Kowluru RA (2012). Damaged mitochondrial DNA replication system and the development of diabetic retinopathy. Antiox Redox Signal..

[17] Mishra M, Kowluru RA (2014). Retinal mitochondrial DNA mismatch repair in the development of diabetic retinopathy, and its continued progression after termination of hyperglycemia. Invest. Ophthalmol. Vis. Sci..

[18] Madsen-Bouterse SA, Mohammad G, Kanwar M, Kowluru RA (2010). Role of mitochondrial DNA damage in the development of diabetic retinopathy, and the metabolic memory phenomenon associated with its progression. Antiox Redox Signal..

[19] Holliday R (2006). Epigenetics: a historical overview. Epigenetics.

[20] Liu MM, Chan CC, Tuo J (2013). Epigenetics in ocular diseases. Curr. Genomics.

[21] Kowluru RA (2017). Diabetic retinopathy, metabolic memory and epigenetic modifications. Vis. Res..

[22] Mishra M, Kowluru RA (2019). DNA methylation-a potential source of mitochondria DNA base mismatch in the development of diabetic retinopathy. Mol. Neurobiol..

[23] Mishra M, Kowluru RA (2016). The role of DNA methylation in the metabolic memory phenomenon associated with the continued progression of diabetic retinopathy. Invest. Ophthalmol. Vis. Sci..

[24] Stuppia G (2015). MFN2-related neuropathies: Clinical features, molecular pathogenesis and therapeutic perspectives. J. Neurol. Sci..

[25] Diabetes Control and Complications Trial/Epidemiology of Diabetes Interventions and Complications group (2015). Effect of Intensive Diabetes Therapy on the Progression of Diabetic Retinopathy in Patients with Type 1 Diabetes: 18 Years of Follow-up in the DCCT/EDIC. Diabetes.

[26] Santos JM, Kowluru RA (2013). Impaired transport of mitochondrial transcription factor A (TFAM) and the metabolic memory phenomenon associated with the progression of diabetic retinopathy. Diabetes Metab. Res. Rev..

[27] Santos JM, Kowluru RA (2011). Role of mitochondria biogenesis in the metabolic memory associated with the continued progression of diabetic retinopathy and its regulation by lipoic acid. Invest. Ophthalmol. Vis. Sci..

[28] Ikeda Y (2015). Molecular mechanisms mediating mitochondrial dynamics and mitophagy and their functional roles in the cardiovascular system. J. Mol. Cell Cardiol..

[29] Chen H, Chomyn A, Chan DC (2005). Disruption of fusion results in mitochondrial heterogeneity and dysfunction. TJ. Biol. Chem..

[30] Wallace DC (1999). Mitochondrial diseases in man and mouse. Science.

[31] Collins DW (2016). Association of primary open-angle glaucoma with mitochondrial variants and haplogroups common in African Americans. Mol. Vis..

[32] Stefano GB, Kream RM (2016). Mitochondrial DNA heteroplasmy in human health and disease. Biomed. Rep..

[33] Hainsworth DP (2019). Risk Factors for Retinopathy in Type 1 Diabetes: The DCCT/EDIC Study. Diab Care.

[34] Chen Z (2016). Epigenomic profiling reveals an association between persistence of DNA methylation and metabolic memory in the DCCT/EDIC type 1 diabetes cohort. Proc. Natl Acad. Sci..

[35] Attwood JT, Yung RL, Richardson BC (2002). DNA methylation and the regulation of gene transcription. Cell Mol. Life Sci..

[36] Tewari S, Zhong Q, Santos JM, Kowluru RA (2012). Mitochondria DNA replication and DNA methylation in the metabolic memory associated with continued progression of diabetic retinopathy. Invest. Ophthalmol. Vis. Sci..

[37] Duraisamy AJ, Mishra M, Kowluru RA (2017). Crosstalk between histone and DNA methylation in regulation of retinal matrix metalloproteinase-9 in diabetes. Invest. Ophthalmol. Vis. Sci..

[38] Watson CJ (2016). Epigenetic Therapy for the Treatment of Hypertension-Induced Cardiac Hypertrophy and Fibrosis. J. Cardiovas Pharma Ther..

[39] Song B, Kim D, Nguyen NH, Roy S (2018). Inhibition of Diabetes-Induced Lysyl Oxidase Overexpression Prevents Retinal Vascular Lesions Associated With Diabetic Retinopathy. Invest. Ophthalmol. Vis. Sci..

[40] Kowluru RA, Mishra M, Kowluru A, Kumar B (2016). Hyperlipidemia and the development of diabetic retinopathy: Comparison between type 1 and type 2 animal models. Metabolism.

[41] Kowluru, R. A. Retinopathy in a Diet-Induced Type 2 Diabetic Rat Model, and Role of Epigenetic Modifications. Diabetes (2020) Jan 16 [Epub ahead print]. 10.2337/db19-1009 PMC7085254 31949005

[42] Mohammad G, Duraisamy AJ, Kowluru A, Kowluru RA (2019). Functional Regulation of an Oxidative Stress Mediator, Rac1, in Diabetic Retinopathy. Mol. Neurobiol..

[43] Mishra M, Duraisamy AJ, Kowluru RA (2018). Sirt1- A guardian of the development of diabetic retinopathy. Diabetes.

